# A unimolecular theranostic system with H_2_O_2_-specific response and AIE-activity for doxorubicin releasing and real-time tracking in living cells†

**DOI:** 10.1039/c8ra01185k

**Published:** 2018-03-20

**Authors:** Xiaoying Gao, Jie Cao, Yinuo Song, Xiao Shu, Jianzhao Liu, Jing Zhi Sun, Bin Liu, Ben Zhong Tang

**Affiliations:** MOE Key Laboratory of Macromolecular Synthesis and Functionalization, Department of Polymer Science and Engineering, Zhejiang University Hangzhou 310027 China liujz@zju.edu.cn; Department of Chemical and Biomolecular Engineering, National University of Singapore 4 Engineering Drive 4 Singapore 117585 Singapore cheliub@nus.edu.sg; Department of Chemistry, Division of Biomedical Engineering, The Hong Kong University of Science and Technology Clear Water Bay, Kowloon Hong Kong China tangbenz@ust.hk; SCUT-HKUST Joint Research Laboratory, Guangdong Innovative Research Team, State Key Laboratory of Luminescent Materials and Devices, South China University of Technology Guangzhou 510640 China

## Abstract

A theranostic drug delivery system composed of tetraphenyl-ethene (AIEgen), benzyl boronic ester (trigger), and doxorubicin (drug) was designed and synthesized; its utilities for cell imaging, drug delivery tracking, and cancer cell cytociding were evaluated.

## Abstract 2

A theranostic drug delivery system composed of tetraphenylethene (AIE-gen), benzyl-boronic ester (trigger) and doxorubicin (drug) was synthesized and its functions in cell imaging, drug delivery tracking, and therapeutic effect were evaluated.

## Graphical abstract

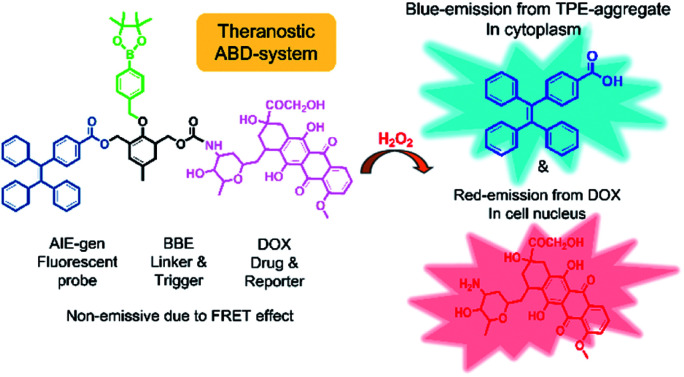

Cancer is a disease that heavily threatens human lives and chemotherapy is known to be an effective route for cancer treatment.^1,2^ To achieve good therapeutic efficiency, controlled drug delivery systems are usually used in chemotherapy. Traditional drug delivery systems are nanocarriers that could specifically respond to a certain physiological environment (*e.g.*, redox, oxidation, pH, *etc.*), and release the drug at the same time.^3–5^ Recently, some theranostic drug delivery systems have been developed and attracted broad attention.^6–9^ Theranostic drug delivery systems that integrate diagnosis and therapy often respond to cancer-associated stimuli, release the drug, and simultaneously reveal an output signal change which can be tracked in a real time manner.^6–9^

Fluorescent probing is one of the mostly used signaling techniques to display signal change upon drug release. Recently, fluorescent probes with AIE (aggregation-induced emission) property, namely AIEgens, have attracted great attention in both fundamental research and industrial applications.^10–16^ AIEgens are non-emissive when molecularly dissolved in solution but can be induced to emit strongly in aggregate state because of the restricted intramolecular motions (RIM).^10–16^ This unique RIM mechanism allows AIEgens to emit efficiently at high concentrations and in solid state. Compared with conventional dyes with aggregation caused quenching (ACQ) property, AIEgens have shown higher photo-bleaching resistance and emission stability. In addition, they have shown lower cytotoxicity than inorganic quantum dots. Thus they are ideal candidates for cell imaging and drug delivery tracking.^13–16^

The application of AIEgens to real-time imaging and tracking drug release process emerged as a novel strategy. For example, Liu *et al.* developed a light-up theranostic agent by tactfully linking a silole-based AIEgen, a cyclic RGD cancer cell-targeting peptide, a platinum Pt(iv) prodrug, and a stimulus responsive peptide together.^17^ The reductant of ascorbic acid in U87-MG cancer cells reduced the prodrug Pt(iv) to the active Pt(ii) drug, which triggered apoptosis by activating the pro-apoptotic enzyme caspase-3. Subsequently, the enzyme cleaved the peptide to release the AIEgens, which aggregated in the cytoplasm and emitted strongly to report the drug-induced apoptosis. Using this strategy, another light-up theranostic agent containing a tetraphenylethene (TPE) AIEgen, a RGD peptide, a Pt(iv) prodrug and a stimulus-responsive peptide was designed, and it showed evident AIE-characteristics and expected cytotoxicity for breast cancer cells over normal cells. In a later design, both prodrugs of Pt(iv) and doxorubicin (DOX) were introduced into the light-up theranostic agent, which enhanced the apoptosis of cancer cells by the synergistic effect of the two drugs.^18^ In 2014, Zou, Liang and colleagues reported a fluorescence based self-indicating drug delivery system, which is capable of signaling spatiotemporal drug release with TPE and DOX composite nanoparticles (NPs).^5^ The emission of TPE molecules in the composite NPs was partially quenched by the DOX aggregates *via* a fluorescence resonance energy transfer (FRET) mechanism. After being taken up into lysosomes, the low internal pH triggered the detachment of DOX from the composite NPs and simultaneously enhanced the emission from AIEgens due to the absence of FRET process. This drug delivery mode can track the sub-cellular location of the delivery system and the drug releasing site.

In this work, we developed a theranostic drug delivery system different from those reported in the literature. The system is constructed from an AIEgen (carboxylated TPE), a benzyl-boronic ester (BBE), and a DOX prodrug. It is abbreviated as ABD-system, and its working mechanism is illustrated in Scheme 1. In comparison with the previous work, a prime difference in our design lies on the BBE trigger, which is sensitive to hydrogen peroxide that is one of the cellular reactive oxygen species (ROS). ROS play key roles in controlling the function and health of cells, and alleviated ROS level has been correlated to cancerous cells.^19–24^ With their high reactivity, several drug delivery systems utilized ROS-sensitive functional groups, such as BBE and 2,4-dinitro-benzene sulfonyl, as triggers to activate prodrugs.^25–28^ So far, cellular ROS has never been used as the internal agent to trigger the drug release during which a fluorescence turn-on mechanism is utilized to monitor the progress in a real-time mode. When chemically bonded together *via* a self-immolative BBE linker,^29^ the TPE and DOX units are close to each other. Moreover, the emission maximum (480 nm) of TPE is largely over-lapped with the absorption maximum (480 nm) of DOX. As DOX is weakly emissive due to its ACQ property, the ABD system is faintly emissive in solution or in aggregate state because of the unique emission mechanism of the AIEgen and the efficient FRET from TPE to DOX. If the ABD systems are internalized into a living cell with elevated cellular ROS level, the BBE linker would be cleaved, thereby the DOX and TPE units would be disassociated with each other and the FRET process is disrupted. As a result, DOX would be released and taken up into cell nucleus with red fluorescence. Meanwhile, the TPE molecules would form aggregate in the cellular plasma for their hydrophobic nature thereby the blue fluorescence could be detected. By monitoring the fluorescence changes in blue and red channels, the spatial and temporal information of the DOX release could be extracted.

**Scheme 1:**
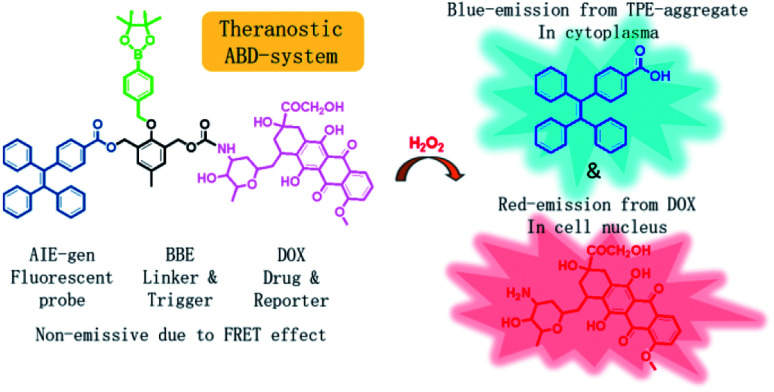
Chemical structure of the theranostic drug delivery ABD-system and a schematic illustration of its working mechanism in a cell with elevated hydrogen peroxide level.

The chemical structure of the theranostic ABD-system is shown in Scheme 1, and its synthetic route, preparation procedure, and characterization data of related intermediates are described in the Experiment section and ESI† (Fig. S1 to S12†). The spectral characterization results indicated that the expected ABD-system has been successfully obtained. With the ABD-system in hand, we first investigated its fluorescence responses towards hydrogen peroxide H_2_O_2_, in order to examine the proposed working mechanism shown in Scheme 1. The ABD-system itself in the solid state was weakly red emissive under the excitation of UV light due to efficient FRET, in spite of the strong emission of the TPE unit in solid state. In PBS buffer, almost no emission was recorded for the ABD-system upon excitation at 330 nm (Fig. 1), because the fluorescence of TPE is absorbed by DOX, while DOX is weakly fluorescent in aggregate state (the size distribution of the aggregates is shown in Fig. S13†). After the addition of H_2_O_2_ (100 μM) into buffer solution, blue fluorescence was observed and the brightness enhanced gradually with time. As shown in Fig. 1A and B, the emission peak appeared at around 480 nm, which was assigned to the emission from the aggregates of hydrophobic carboxylated TPE molecules, as indicated by its AIE property (Fig. S15 and S16†). This is because, when the BBE unit is oxidized by H_2_O_2_, the linkage between TPE and DOX is broken, and DOX and carboxylated TPE are released (*cf.* Fig. S17†). The hydrophobic carboxylated TPE molecules form aggregates which emit blue fluorescence, while the DOX molecules are soluble in the buffer solution and emit weak red fluorescence. Since the distance between carboxylated TPE and DOX is far enough, FRET process is prohibited and hence the blue emission has been observed.

**Fig. 1:**
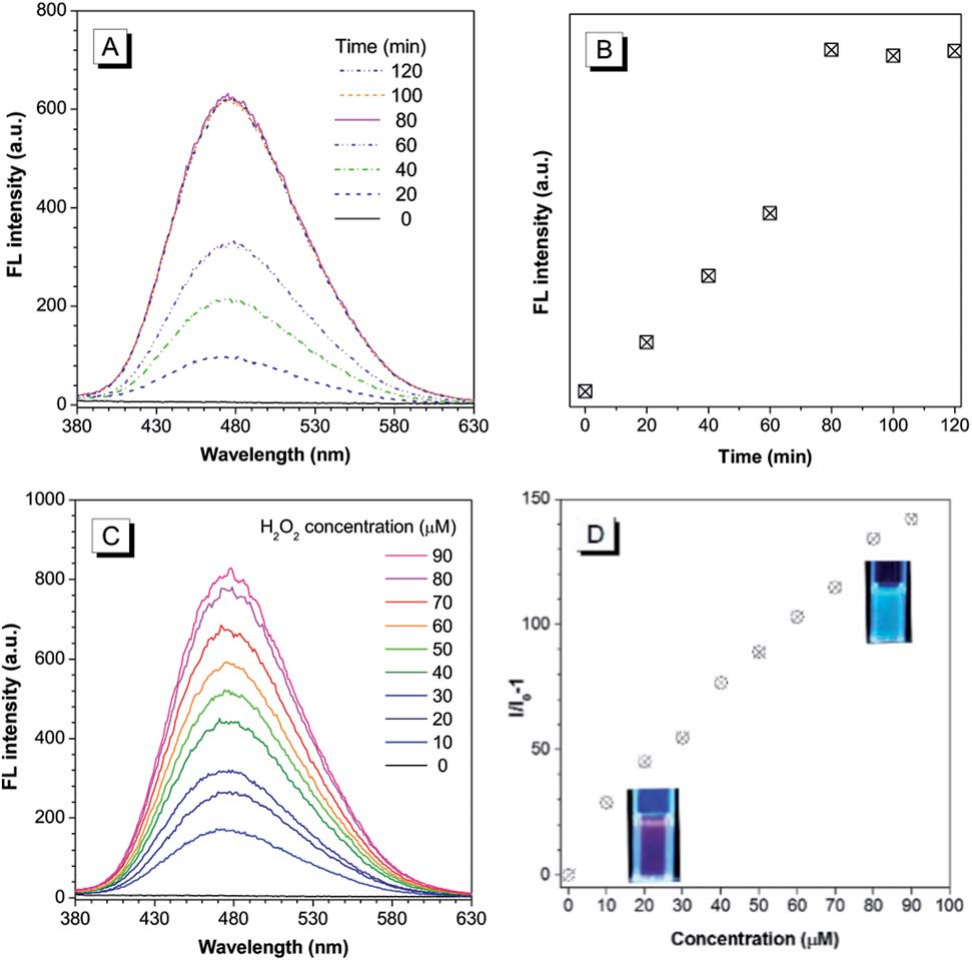
(A) Changes in fluorescence (FL) spectra of the ABD-system (10 μM) in PBS buffer solution in the presence of H_2_O_2_ (100 μM) for different times (0 to 90 min). (B) Plot of FL intensity *versus* incubation time. The data are extracted from the spectra in (A). (C) FL spectra of the ABD-system in PBS buffer solution incubated with different concentrations of H_2_O_2_ for 90 min; (D) plot of the relative FL intensity *versus* H_2_O_2_ concentration. The data are extracted from (C), and the inset photographs show the FL images of the stock solutions with and without H_2_O_2_. Concentration of the ABD-system (10 μM); PBS buffer solution (pH = 7.8, 10 mM, containing 1% DMSO); temperature: 37 °C; excitation wavelength: 330 nm for FL measurement and 365 nm for photograph.

As indicated by the experimental data shown in Fig. 1A and B, the emission intensity increased gradually and the spectral features reached a steady state (unchanged) after 80 min. The experimental results shown in Fig. 1C and D were obtained by reacting with H_2_O_2_ for 90 min. A prominent dose-dependent behavior is observed. The emission intensity continuously increases with the increasing H_2_O_2_ concentration from 10 to 90 μM, and the photograph clearly displays the blue emission from the AIEgen (inset of Fig. 1D). A fluorescence intensity enhancement of 142-fold was observed when the H_2_O_2_ concentration was increased to 90 μM.

Considering that other ROS, RNS (reactive nitrogen species) and RSS (reactive sulphur species), such as O_2_^−^, GSH, ClO^−^, ·OH, ^1^O_2_, *tert*-butyl hydroperoxide (TBHP), alkylperoxyl radical (ROO·), NO_2_^−^, NO_3_^−^, and S_2_^−^, may coexist with H_2_O_2_ in physiological environment, we also examined the fluorescent response of the ABD-system to the interfering species. As shown in Fig. 2, except for H_2_O_2_, all of the other reactive species can only lead to very small changes in fluorescence intensity under the identical experimental condition. Such an excellent selectivity is ascribed to the rational molecular design, since among the ROS we tested, only H_2_O_2_ can react with the BBE unit to induce the separation between TPE and DOX units and then turn on fluorescence emission.

**Fig. 2:**
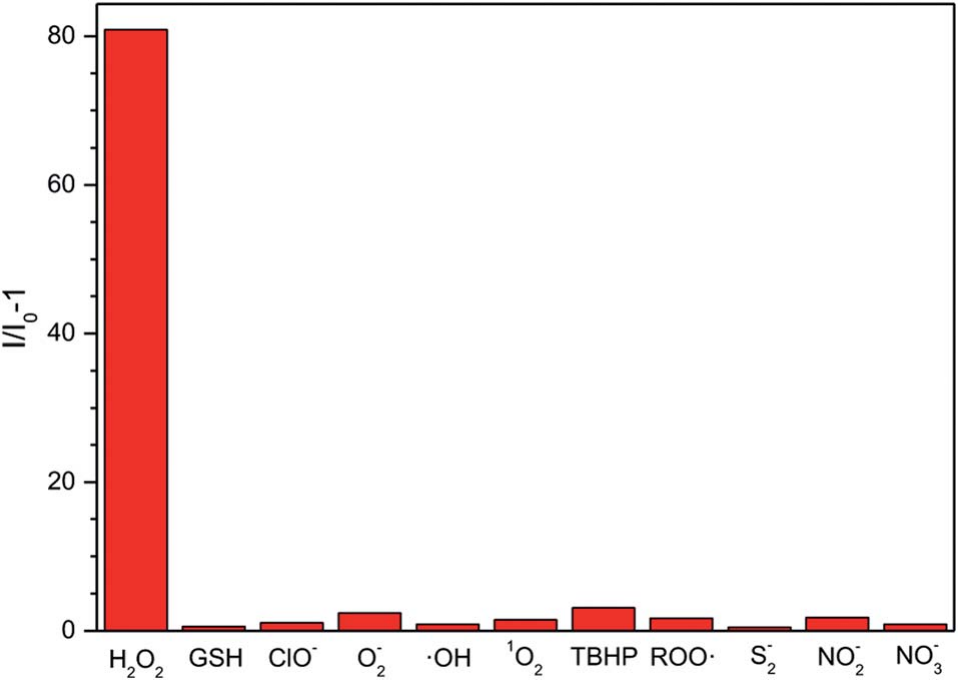
FL response of the ABD system (10 μM) to a series of ROS (100 μM) in PBS buffer solution. *I*_0_ and *I* are the FL intensity of the ABD-system in PBS buffer solution in the presence of ROS, RNS, and RSS including H_2_O_2_, O_2_^−^, GSH, ClO^−^, ·OH, ^1^O_2_, TBHP, ROO·, S_2_^−^, NO_2_^−^ and NO_3_^−^. PBS buffer solution (pH = 7.8, 10 mM, containing 1% DMSO); temperature: 37 °C; excitation wavelength: 330 nm; reaction time: 100 min.

Based on the above results, the ABD system works well in buffer solutions. The blue emission from the aggregate of carboxylated TPE can be specifically triggered by H_2_O_2_, indicating the releasing of DOX. To check whether this system can work well in living cells, we incubated HeLa cells in different conditions and monitored the fluorescence from the cells using confocal laser scanning microscopy (CLSM) technique. When HeLa cells were loaded with 10 μM ABD-system and incubated at 37 °C for 2 h, no fluorescence emission could be recorded in blue channel (420–540 nm), but red emission with moderate intensity was recorded in the spectral window of 550–650 nm. The overlay (Fig. 3D) of the bright field (Fig. 3C) with the fluorescent images suggest that the ABD-system is uniformly distributed in the cytoplasm.

**Fig. 3:**
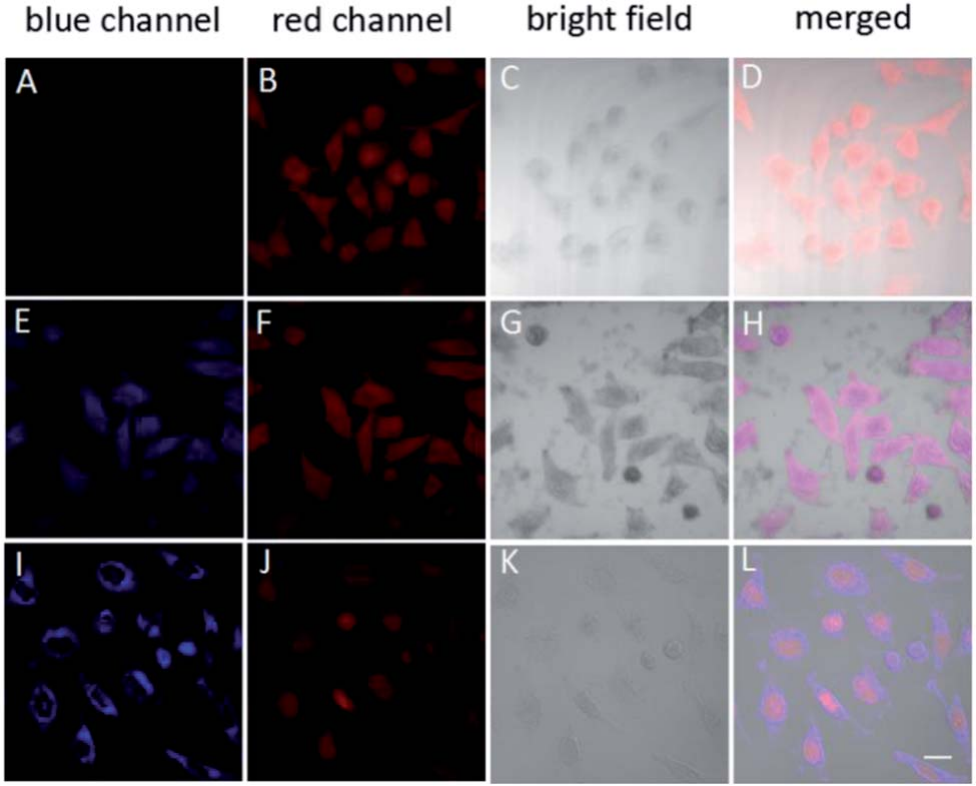
Confocal images of HeLa cells after incubation with TPE–DOX with different treatment; (A–D) no more treatment; (E–H) cells treated with PMA for 30 min and incubated for another 2.5 hour; (I–L) cell treated with PMA for 30 min and incubated for another 5.5 hour. Blue channel: excitation wavelength: 405 nm; collection wavelength: 420–540; red channel: excitation wavelength: 488 nm; collection wavelength: 550–650 nm. All images share the same scale bar (20 mm).

When ABD-system loaded HeLa cells were treated with 5 μg mL^−1^ phorbol-12-myristate-13-acetate (PMA) for 30 min and then incubated for another 2.5 hours, evident blue emission was detected in spectral window of 420–540 nm (Fig. 3E). Meanwhile, red emission was also collected in the wavelength range of 550–650 nm (Fig. 3F). Since PMA is an agent widely used to *in situ* induce the generation of H_2_O_2_ in living cells, the introduction of PMA can effectively elevate the H_2_O_2_ level and thereby trigger the release of both carboxylated TPE and DOX in the cytoplasm. The generated carboxylated TPE molecules were insoluble in aqueous medium (*e.g.*, cytoplasm), and hence formed aggregates. Due to the inhibited FRET process induced by DOX leaving and the TPE aggregate formation, strong blue fluorescence from TPE donor was recorded according to the RIM mechanism. At the same time, DOX molecules were also released into cytoplasm. The merged image indicates that the blue and red emissions from the aggregates of the carboxylated TPEs and DOXs have good overlap and are distributed all over the cytoplasm.

After further incubation for additional 3 h, the blue emission still existed in the cytoplasm (Fig. 3I), while the red emission could be observed in the cell nucleus (Fig. 3J). This indicates that some DOX molecules are translocated into cell nucleus. This observation is reasonable because DOX is a drug that works on DNA. In the overlay image (Fig. 3L), the entities with purple fluorescence are assigned to cell nucleus, indicative of the colour mixing of blue and red emissions. The surrounding blue fluorescence comes from the aggregated carboxylated TPEs.

In order to evaluate therapeutic performance of the ABD system, cell viability was studied using MTT (3-(4,5-dimethyl-thiazol-2-yl)-2,5-diphenyltetrazolium bromide) assay. As shown in Fig. 4, without the ABD system, the cell viability is as high as 98% in the presence of PMA under standard incubation condition. PMA itself has no therapeutic effect on HeLa cells. Without the treatment of PMA, the presence of ABD system could not induce the therapeutic effect, and the cell viabilities in different concentrations and incubation time were almost the same, as suggested by the data in Fig. 4. In sharp contrast, when activated with PMA, cell viabilities decreased dramatically and synergistically with the increment of prodrug concentration and incubation time. As a direct evidence, the bright field images (Fig. 3C, G and K) and the confocal fluorescent images demonstrate that the morphologies of HeLa cells changed significantly upon treated with ABD and PMA for hours. These results strongly prove that ABD system has good therapeutic effects on HeLa cells.

**Fig. 4:**
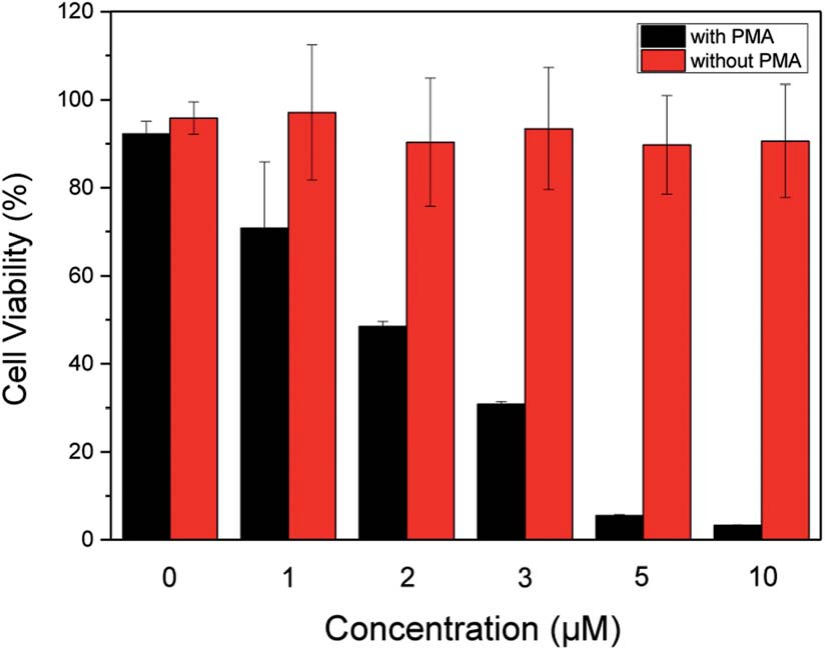
Dose-dependent cytotoxicity of ABD system to HeLa cells with and without PMA treatments, which were estimated by using standard MTT assay.

In summary, an ABD-system consisting of carboxylated TPE (AIEgen), active linker (benzyl-boronic ester) and DOX (drug) moieties has been designed, synthesized and characterized. Its drug releasing and fluorescent tracking processes and its therapeutic effect have been studied. In living HeLa cells incubated with the ABD-system, sole red emission can be observed. After treatment with PMA, H_2_O_2_ is generated *in situ* and it reacts with the benzyl-boronic ester moiety, leading to the decomposition of the ABD system and the spatial separation of carboxylated TPE and DOX moieties. As a result, both of the blue emission from the AIEgen and red emission from DOX molecules have been monitored in the respective spectral channels, due to restricted FRET between AIEgens and DOX. Based on the dual emission colors, the DOX releasing can be monitored. After a short period of time, the released DOX molecules enter into nucleus to realize its therapeutic function, which is revealed by the observation of the red emission in the nucleus region. The therapeutic effect has been estimated by the MTT experiments. In addition, the ABD system shows high stability in the pH range from 4 to 9 and good selectivity over other interfering reactive species including OCl^−^, O_2_^−^, ·OH, GSH, ^1^O_2_, TBHP, ROO·, NO_2_^−^, NO_3_^−^ and S_2_^−^, which are also involved in biological systems.

## Conclusions

In short, the ABD system demonstrates multiple functions of fluorescent cell imaging, drug releasing and drug delivery process tracking, and thus it is a novel and promising candidate for theranostic drug delivery system.

## Conflicts of interest

There are no conflicts to declare.

## Supplementary Material

RA-008-C8RA01185K-s001
