# Supplementary material for: A unimolecular theranostic system with H2O2-specific response and AIE-activity for doxorubicin releasing and real-time tracking in living cells
Source: RSC Adv. 2018 Mar 20;8(20):10975–9. doi: 10.1039/c8ra01185k (PMC9078942; doi:10.1039/c8ra01185k)
Supplement: RA-008-C8RA01185K-s001 [file RA-008-C8RA01185K-s001.pdf]

Electronic Supplementary Material (ESI) for RSC Advances.
This journal is © The Royal Society of Chemistry 2018

Electronic Supplementary Information

A unimolecular theranostic system with H_2O_2-specific response and AIE-activity for doxorubicin releasing and real-time tracking in living cells

Xiaoying Gao,[a] Jie Cao,[a] Yinuo Song,[a] Xiao Shu,[a] Jianzhao Liu,*[a] Jing Zhi Sun,[a] Bin Lin,[b*] and Ben Zhong Tang*[acd]

[a] MOE Key Laboratory of Macromolecular Synthesis and Functionalization, Department of Polymer Science and Engineering, Zhejiang University, Hangzhou 310027, China

[b] Department of Chemical and Biomolecular Engineering, National University of Singapore, 4 Engineering Drive 4, Singapore, 117585 (Singapore)

[c] Department of Chemistry, Division of Biomedical Engineering, The Hong Kong University of Science and Technology, Clear Water Bay, Kowloon, Hong Kong (China)

[d] SCUT-HKUST Joint Research Laboratory, Guangdong Innovative Research Team, State Key Laboratory of Luminescent Materials and Devices, South China University of Technology, Guangzhou, 510640 (China)

Materials and Instrumentation and cell culture

Materials. All the chemicals and reagents are commercially available and used without further purification. DCM, THF, DMF, acetone and methanol are distilled under nitrogen before use. 2,6-Bis(hydroxymethyl)-*p*-cresol, (1,1-Dimethylethyl) dimethylsilyl chloride, imidazole, (4-Bromomethylphenyl)boronic acid pinacol ester, cesium carbonate, *p*-toluenesulfonic acid, 1-Ethyl-3-(3-dimethylaminopropyl) carbodiimide hydrochloride (EDC), 4-dimethylaminopyridine (DMAP) and 4-nitrophenyl chlorocarbonate are purchased from TCI. Triethylamine, phorbol-12-myristate-13-acetate (PMA), doxorubicin (DOX) are purchased from Sigma Aldrich. Fetal bovine serum (FBS) and 3-(4,5-dimethylthiazol-2-yl)- 2,5-diphenyltetrazolium bromide (MTT) are purchased from Invitrogen. PBS buffer (pH = 7.4, 10 mM) is prepared with pure water from a Millipore filtration system.

Instrumentation. ^{1}H and ^{13}C NMR spectra were measured on Bruker ARX 400 NMR spectrometer using DMSO as solvent, and tetramethylsilane (TMS; δ = 0 ppm) was chosen as internal reference. Photoluminescence and UV spectra were measured on PerkinElmer LS 55 spectrofluorometer and Biochrom Libra S80PC double beam spectrometer, respectively. High-resolution mass spectra (HRMS) are measured on a GCT Premier CAB 048 mass spectrometer operating in MALDI-TOF mode. Particle sizes are measured using a Brookhaven ZetaPlus potential analyzer (Brookhaven instruments corporation, USA). Confocal lasing scanning microscopic (CLSM) images and fluorescence spectra are obtained on confocal microscope (Zeiss Laser Scanning Confocal Microscope; LSM7 DUO) using ZEN 2009 software (Carl Zeiss).

Sample preparation. The stock solution of probe was prepared at 1.0 mM in dimethyl sulfoxide (DMSO). Solution of GSH (1.0 mM), NaClO (1.0 mM), H_2O_2 (1.0 mM), $FeSO_4$ (1.0 mM) were prepared with H_2O. KO_2 was used as a solution (1.0 mM) in DMSO. The hydroxyl radical was produced by mixing H_2O_2 (20 μM) with Fe^{2+} (200 μM). Singlet oxygen ($^1O_2$) was obtained by addition H_2O_2 (50 μM) to NaOCl (50 μM). TBHP was purchased from Sigma-Aldrich. $ROO^{\cdot}$ was generated from AAPH.

Titration. Different concentrations of H_2O_2 were added to PBS buffer (0.9 mL) and probe (10.0 μL, 10.0 μM) in a 1.5 mL centrifugal tube, the mixture was added to 1.0 mL by H_2O, containing 1% DMSO. The mixture was vortex shaded and placed at 25 ^oC for 90 min before photoluminescence (PL) spectra was measured. The excitation wavelength was 330 nm.

Cell Culture and Fluorescence Imaging. Hela cells were provided by American Type Culture Collection (ATCC). They were cultured in Dulbecco's Modified Eagle Medium (DMEM) containing penicillin (100 U/mL), 10% heat-inactivated fetal bovine serum (FBS), and streptomycin (100 μg/mL) and were maintained in humidified incubator at 37 ^oC under 5% CO_2 environment. The cells were incubated with 10 μM probe at 37 ^oC for 2 h. After that, the medium was removed and the cells were rinsed with PBS. The probe-loaded cells were treated with PMA for 30min, and incubate for 2 hours and 6 hours, respectively. After that, the media was removed and washed with PBS buffer. The cells were imagined by confocal laser scanning microscope. The excitation wavelength was 405 nm and 488 nm, and the emission filter was 420-540 nm and 560-640 nm.

Cytotoxicity assay. Hela cells were provided by American Type Culture Collection (ATCC). They were cultured in Dulbecco's Modified Eagle Medium (DMEM) containing penicillin (100 U/mL), 10% heat-inactivated fetal bovine serum (FBS), and streptomycin (100 μg/mL) and were maintained in humidified incubator at 37 ^{o}C under 5% CO_2 environment. After removal of the medium, cells were incubated with various concentrations of TPE-DOX for 1h. TPE-DOX loaded cells are treated with and without PMA for 30 min. Then medium was replaced with fresh DMEM and cells continue to be incubated for 48 hours, respectively. The cytotoxicity of the probe was assessed by MTT assay according to ISO 10993-5. For each independent experiment, the assays were performed in five replicates. And the statistic mean and standard derivation were utilized to estimate the cell viability.

2. **Synthetic procedures**

Compound 1 and compound 3 were synthesized according to the procedures reported in literature.[1,2] Compound 2 was purchased from AIEgen Biotech Co., Limited.

Synthesis of compound 1. 2,6-bis(hydroxymethyl)-4-cresol (1.7 g, 10 mmol) and 4-bromomethylphenylboronic acid pinacol ester (2.97 g, 10 mmol) dissolved in dry DMF (50 mL) are stirred for 12 h at 70 ^{o}C. The crude product was extracted with DCM for 3 times and purified by silica gel chromatography with hexane/ethyl acetate (2:1) as eluent to afford the desired product as white solid. Yield = 40%. ^{1}H NMR (400 MHz, DMSO): δ = 7.74-7.70 (d, 2H), 7.50-7.46 (d, 2H), 7.155 (s, 2H), 5.105- 5.055 (t, 2H), 4.84 (s, 2H), 4.525-4.485 (d, 4H), 2.294 (s, 3H), 1.312 (s, 12H) ppm; ^{13}C NMR (100 MHz, DMSO): δ = 150.9, 140.9, 134.7, 134.5, 132.6, 127.6, 127.1, 83.7, 75.3, 57.9, 24.7, 20.8 ppm; HRMS (ESI, m/z), calcd.: 384.21, found: 407.2.

Synthesis of compound 3. Compound 2 (185 mg, 0.5 mmol) and EDC (115 mg, 0.6 mmol) dissolved in dry DCM (50 mL) were stirred for 1 h at 0 ^{o}C. DMAP (13 mg, 0.1

mmol) and compound 1 (226 mg, 0.6 mmol) was added dropwise at 0 ^{o}C. The reaction was stirred at room temperature overnight. The crude product was extracted with DCM for 3 times and purified by silica gel chromatography with hexane/ethyl acetate (2:1) as eluent to afford the desired product as white solid. Yield = 70%. ^{1}H NMR (400 MHz, DMSO):δ = 7.71-7.64 (m, 4H), 7.40 (d, 2H), 7.195-7.075 (m, 3H), 6.995-6.945 (m, 6H), 5.267 (s, 2H), 5.192-5.150 (t, 1H), 4.900 (s, 2H), 4.565-4.535 (d, 2H), 2.294 (s, 3H), 1.312 (s, 12H) ppm; ^{13}C NMR (100 MHz, DMSO):δ = 165.2, 152.3, 148.5, 142.7, 142.6, 142.5, 142.0, 140.5, 139.5, 139.2, 135.5, 134.5, 133.2, 131.0, 130.7, 130.6, 129.8, 129.5, 128.7, 128.3, 127.9, 127.8, 127.4, 127.1, 126.9, 126.8, 124.9, 83.7, 75.8, 73.5, 61.9, 57.9, 57.8, 34.3, 30.8, 26.3, 24.9, 24.7, 21.0, 20.5 ppm; HRMS (ESI, m/z), calcd. for [$C_{49}H_{47}BO_6$]: 742.35, found: 765.3362.

Synthesis of compound 4. To a solution of compound 3 (100 mg, 0.135 mmol) and triethylamine (28 mg, 0.27 mmol) in distilled DCM (20 mL), 4-nitrophenyl chlorocarbonate (41 mg, 0.2025 mmol) was added under nitrogen at 0 ^{o}C. The reaction was stirred at room temperature overnight. The reaction was quenched with saturated ammonium chloride solution. The crude product was extracted with DCM for 3 times and purified by silica gel chromatography with hexane/ethyl acetate (4:1) as eluent to afford the desired product as white solid. Yield = 70%. ^{1}H NMR (400 MHz, DMSO): δ = 8.287 (d, 2H), 7.72 (d, 2H), 7.662 (d, 2H), 7.481 (d, 2H), 7.420 (d, 2H), 7.362 (d, 2H), 7.195-7.085 (m, 11H), 7.000-6.950 (m, 6H), 5.350-5.318 (m, 4H), 4.978 (s, 2H), 2.327 (s, 3H), 1.308 (s, 12H) ppm; ^{13}C NMR (100 MHz, DMSO): δ = 165.27, 165.22, 163.91, 157.55, 155.23, 155.19, 153.54, 153.31, 151.89, 151.82, 148.52, 145.12, 142.66, 142.58, 142.44, 142.2, 139.57, 139.51, 139.12, 134.54, 133.68, 131.04, 130.6, 130.55, 129.99, 129.44, 129.35, 128.78, 128.1, 128.0, 127.92, 127.88, 127.79, 127.32, 126.93, 126.86, 126.75, 126.12, 125.32, 125.26, 124.88, 122.48, 122.43, 115.75, 115.17, 83.63, 81.32, 76.85, 65.90, 34.12, 30.37, 24.62, 24.42, 20.99, 20.31 ppm; HRMS (ESI, m/z), calcd. for [$C_{56}H_{50}BNO_{10}$]: 907.35, found: 930.3436.

Synthesis of ABD system. DOX (13 mg, 0.022 mmol) and triethylamine (7 mg, 0.066 mmol) was stirred in dry DMF (5 mL) at 0 ^{o}C for 1 hour. Compound 4 (20 mg, 0.022

mmol) was added dropwise under 0 °C. The solution was stirred for 4 hours. The reaction was quenched with saturated ammonium chloride solution. The crude product was extracted with DCM for 3 times and concentrated by rotary evaporator with water base off. The crude product was purified by HPLC and lyophilized under vacuum to yield red powders. Yield = 40%. ^{1}H NMR (400 MHz, DMSO): δ = 7.92 (d, 2H), 7.69-7.6 (m, 4H), 7.57 (d, 2H), 7.22 (d, 2H), 7.18-7.05 (m, 12H), 7.0-6.91 (m, 8H), 5.48 (s, 1H), 5.27-5.20 (m, 3H), 5.02-4.84 (m, 6H), 4.58 (s, 2H), 4.16 (d, 1H), 3.98 (s, 3H), 3.72 (s, 1H), 3.0 (d, 2H), 2.24 (s, 3H), 2.16 (d, 2H), 1.86 (t, 1H), 1.47 (d, 1H), 1.28 (s, 12H), 1.12 (d, 3H) ppm; HRMS (ESI, m/z), calcd. for [$C_{77}H_{74}BNO_{18}$]: 1311.50, found: 1334.4920.

References

1. C. de G. Lux, S. Joshi-Barr, T. Nguyen, E. Mahmoud, E. Schopf, N. Fomina and A. Almutairi, *J. Am. Chem. Soc.,* 2012, **134**, 15758–15764.

2. Z. Song, Y. Hong, R. T. K. Kwok, J. W. Y. Lam, B. Liu and B. Z. Tang, *J. Mater. Chem. B.,* 2014, **2**, 17172.

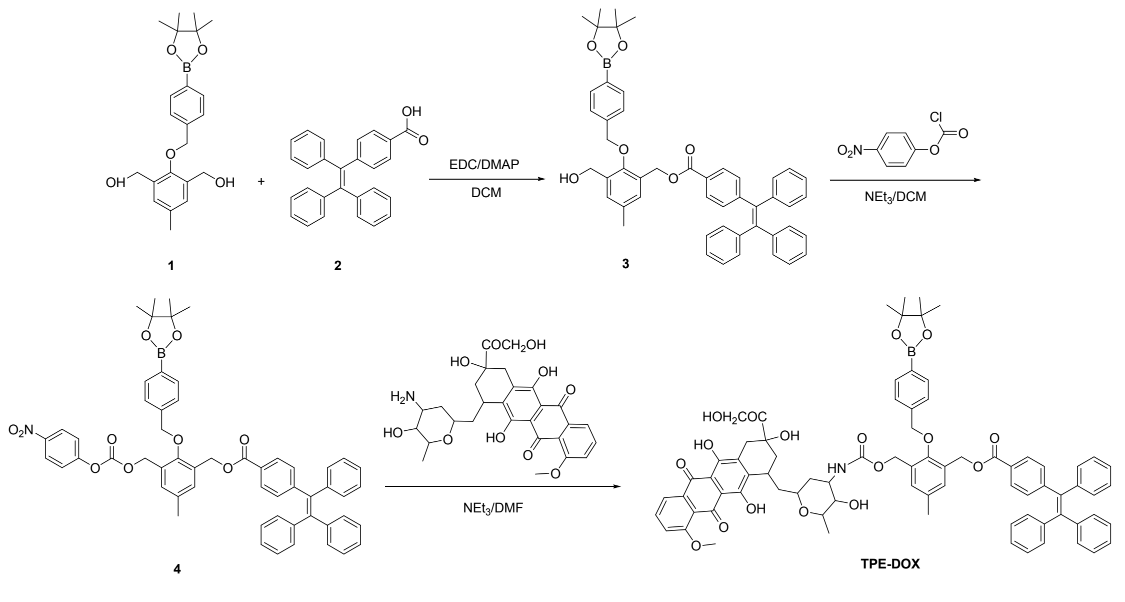

Scheme S1. Synthetic route to the target prodrug TPE-DOX

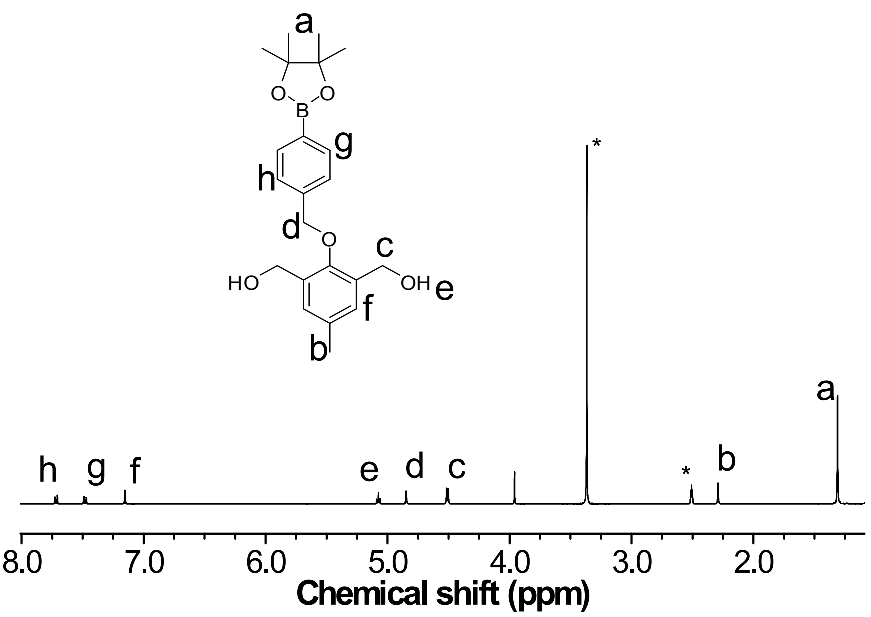

Figure S1. ^{1}H NMR spectrum of compound 1 in DMSO. The solvent peaks are marked with asterisks.

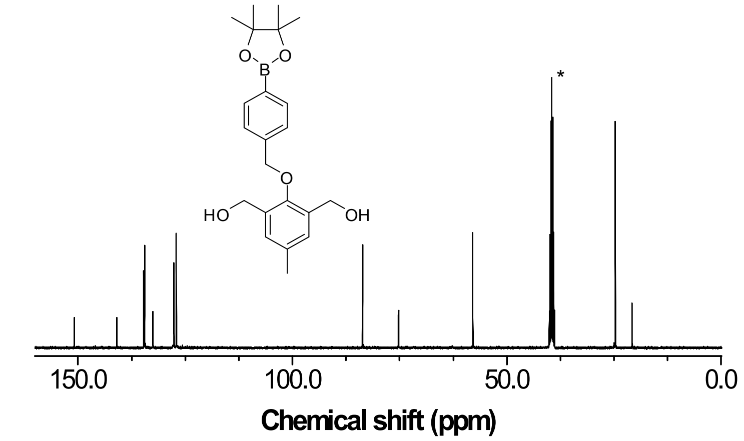

Figure S2. ^{13}C NMR spectrum of compound 1 in DMSO. The solvent peaks are marked with asterisks.

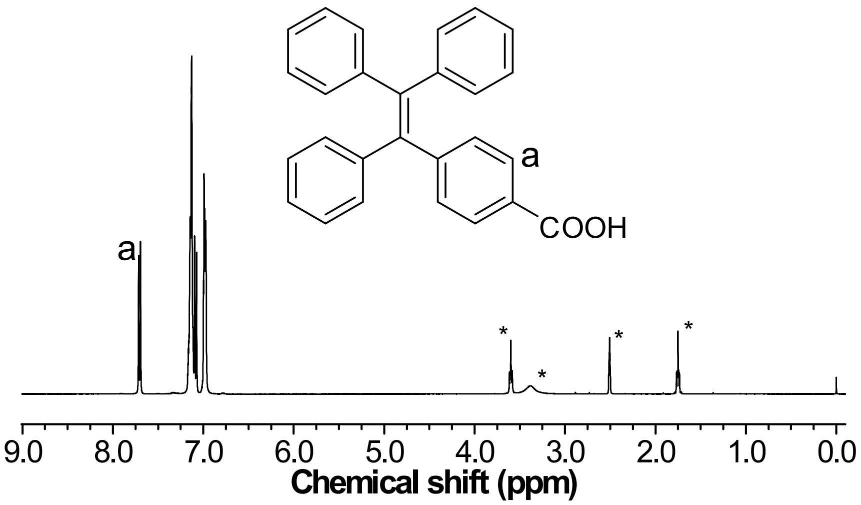

Figure S3. ^{1}H NMR spectrum of compound 2 in DMSO. The solvent peaks are marked with asterisks.

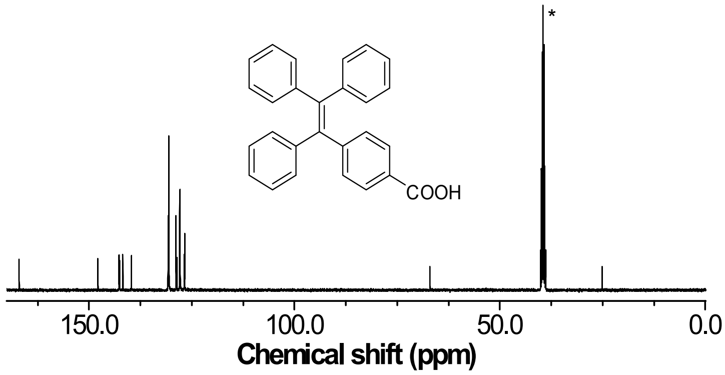

Figure S4. ^{1}H NMR spectrum of compound 2 in DMSO. The solvent peaks are marked with asterisks.

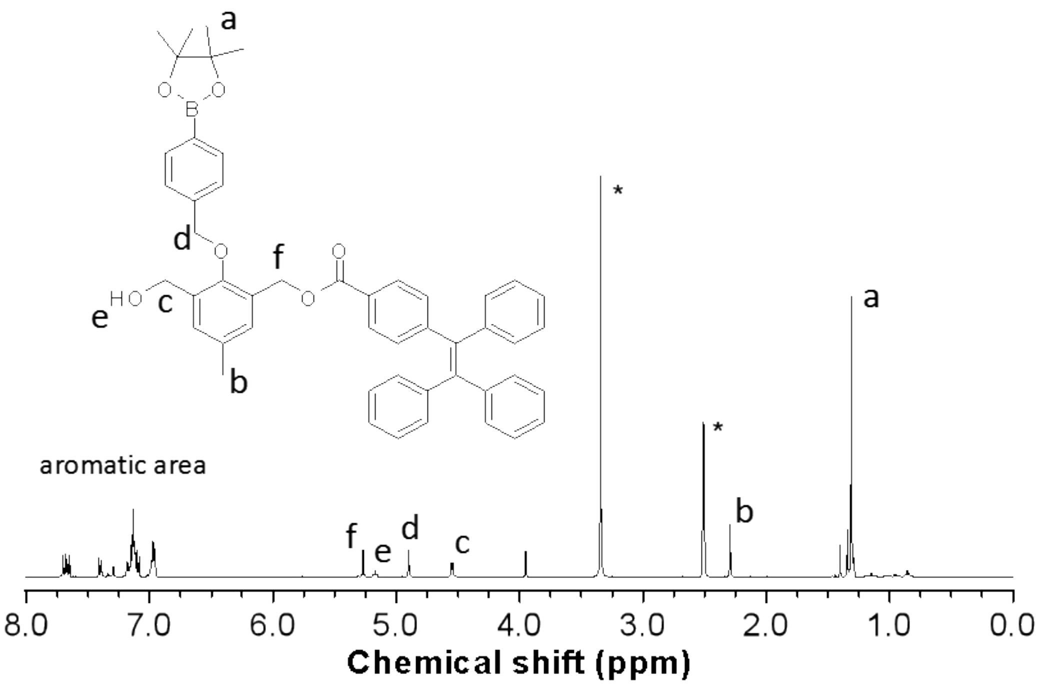

Figure S5. ^{1}H NMR spectrum of compound 3 in DMSO. The solvent peaks are marked with asterisks.

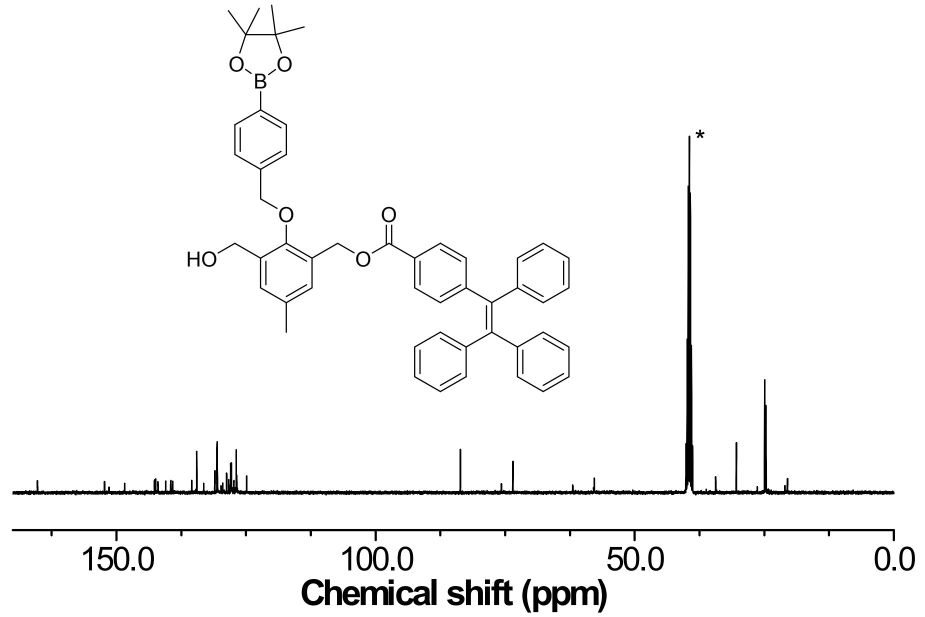

Figure S6. ^{13}C NMR spectrum of compound 3 in DMSO.The solvent peaks are marked with asterisks.

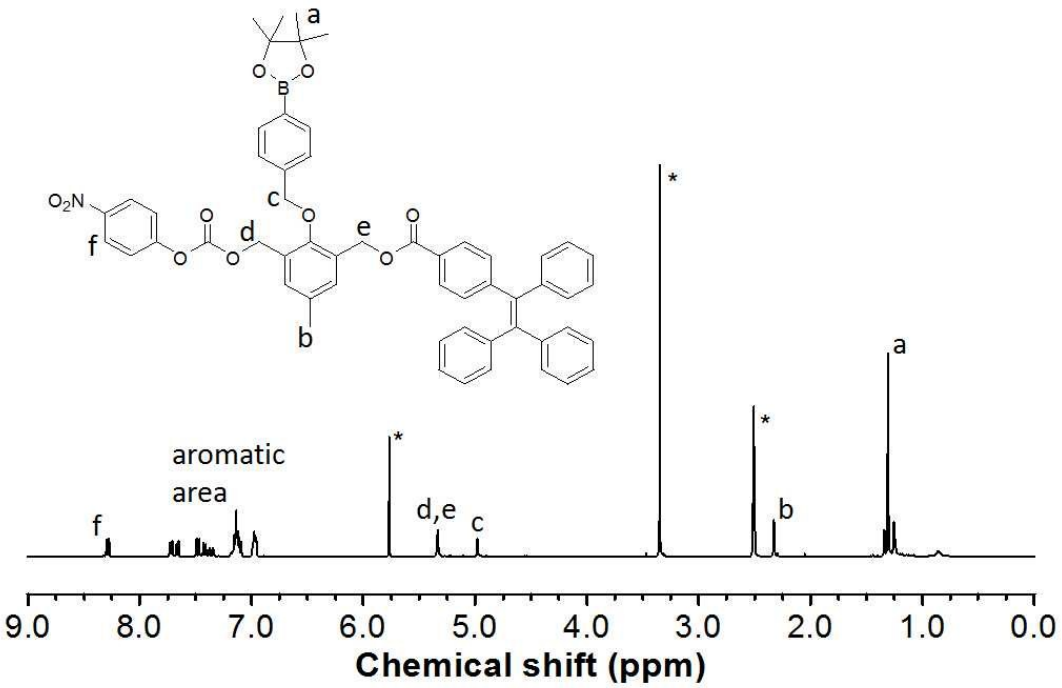

Figure S7. ^{1}H NMR spectrum of compound 4 in DMSO. The solvent peaks are marked with asterisks.

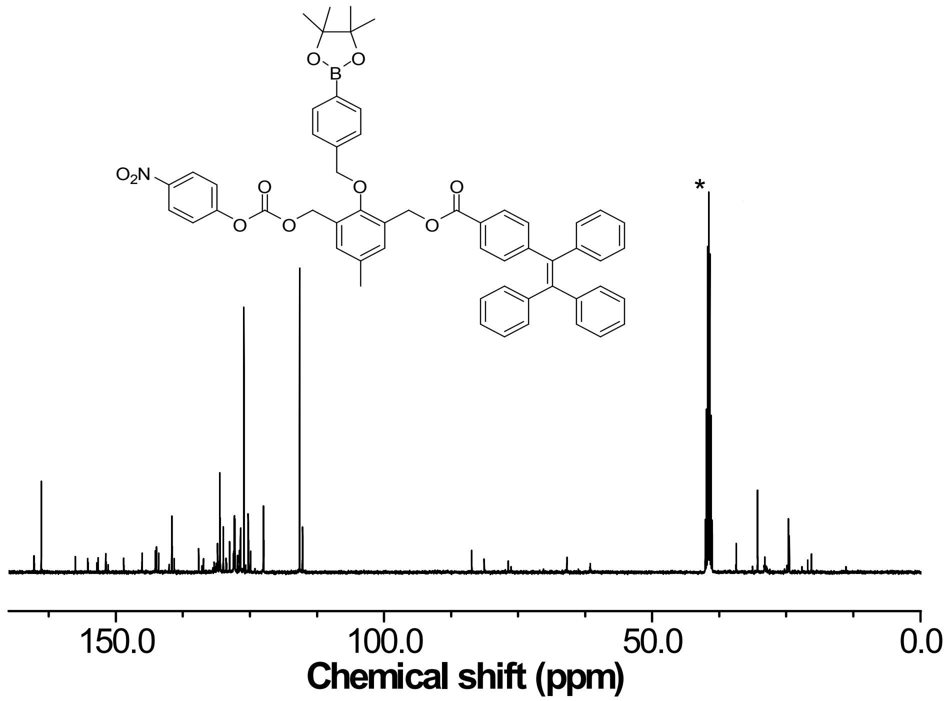

Figure S8. ^{13}C NMR spectrum of compound 4 in DMSO. The solvent peaks are marked with asterisks.

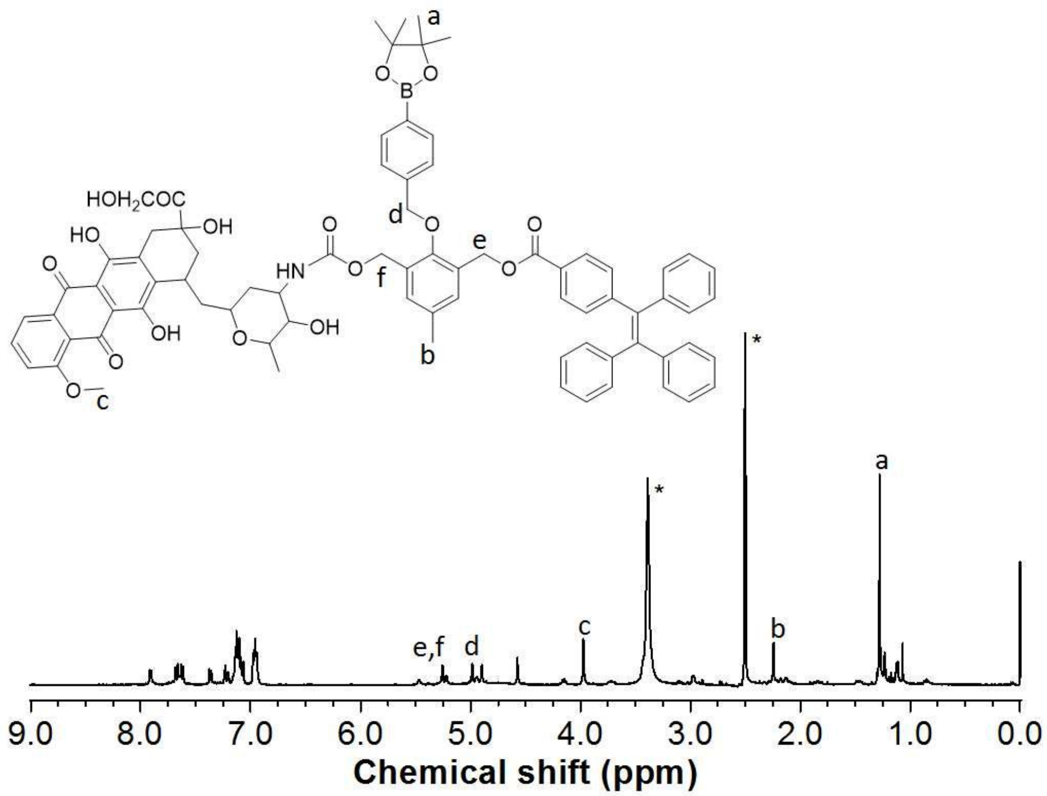

Figure S9. ^{1}H NMR spectrum of ABD system in DMSO-d6. The solvent peaks are marked with asterisks.

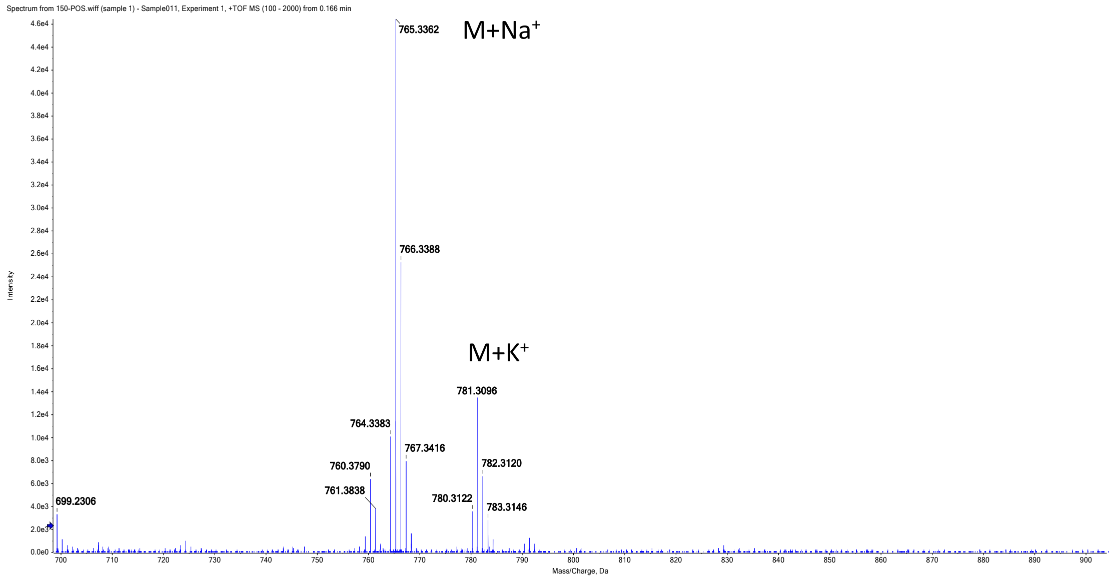

Figure S10. High resolution mass spectrum (HRMS) of compound 3.

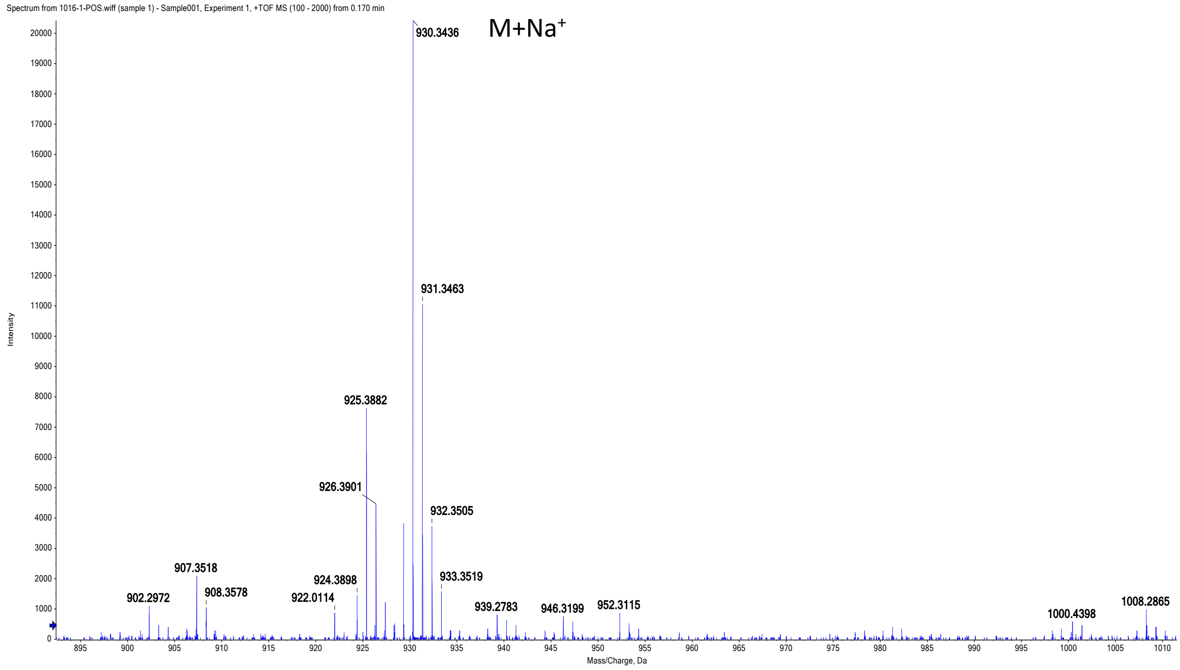

Figure S11. HRMS of compound 4.

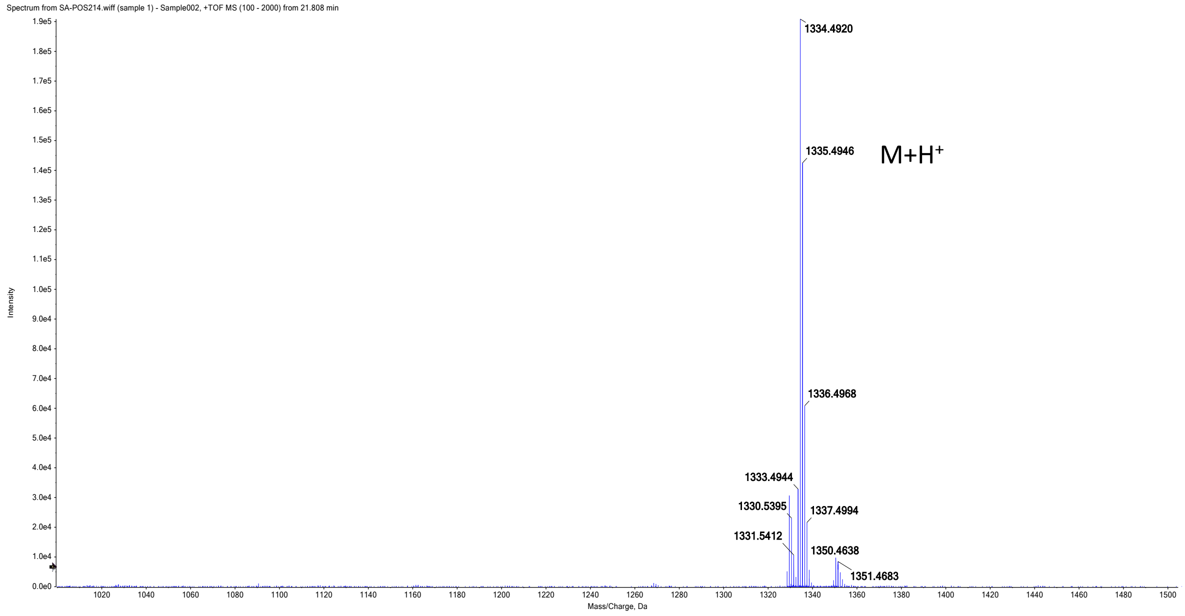

Figure S12. HRMS of ABD system.

Size d.nm	Mean Number Percent	Std Dev Number Percent
0.4000	0.0	
0.4632	0.0	
0.5365	0.0	
0.6213	0.0	
0.7195	0.0	
0.8332	0.0	
0.9649	0.0	
1.117	0.0	
1.294	0.0	
1.499	0.0	
1.736	0.0	
2.010	0.0	
2.328	0.0	
2.696	0.0	
3.122	0.0	
3.615	0.0	
4.187	0.0	
4.849	0.0	

Size d.nm	Mean Number Percent	Std Dev Number Percent
5.615	0.0	
6.503	0.0	
7.531	0.0	
8.721	0.0	
10.10	0.0	
11.70	0.0	
13.54	0.0	
15.69	0.0	
18.17	0.0	
21.04	0.0	
24.36	0.0	
28.21	0.0	
32.67	0.0	
37.84	0.0	
43.82	0.0	
50.75	0.0	
58.77	0.0	
68.06	0.0	

Size d.nm	Mean Number Percent	Std Dev Number Percent
78.82	0.0	
91.28	0.0	
105.7	3.1	
122.4	12.1	
141.8	20.8	
164.2	22.6	
190.1	18.0	
220.2	10.6	
255.0	4.3	
295.3	1.1	
342.0	0.1	
396.1	0.0	
458.7	0.1	
531.2	0.3	
615.1	0.8	
712.4	1.4	
825.0	1.7	
955.4	1.5	

Size d.nm	Mean Number Percent	Std Dev Number Percent
1106	1.0	
1281	0.4	
1484	0.1	
1718	0.0	
1990	0.0	
2305	0.0	
2669	0.0	
3091	0.0	
3580	0.0	
4145	0.0	
4801	0.0	
5560	0.0	
6439	0.0	
7456	0.0	
8635	0.0	
1.000e4	0.0	

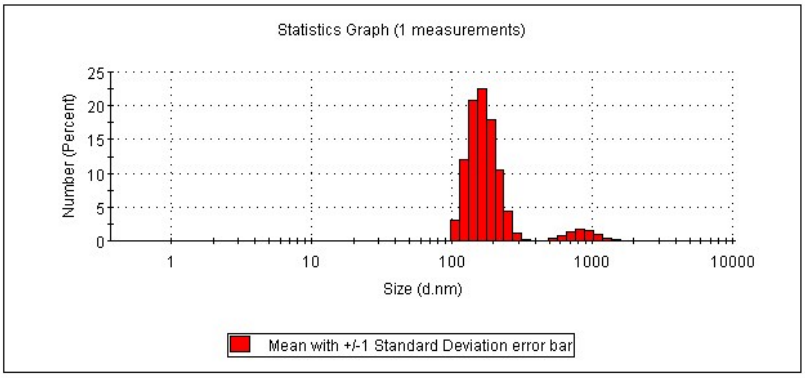

Figure S13. Size distribution of the particles formed by the molecular aggregates of ABD system in PBS buffer containing 1% DMSO at 25 ^{o}C. Concentration: 10 µM.

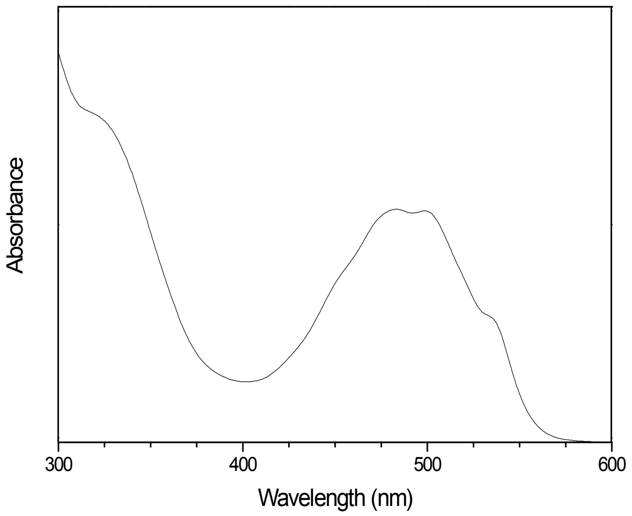

Figure S14. UV-visible spectrum of ABD-system (10 μM) in PBS buffer solution containing 1% DMSO.

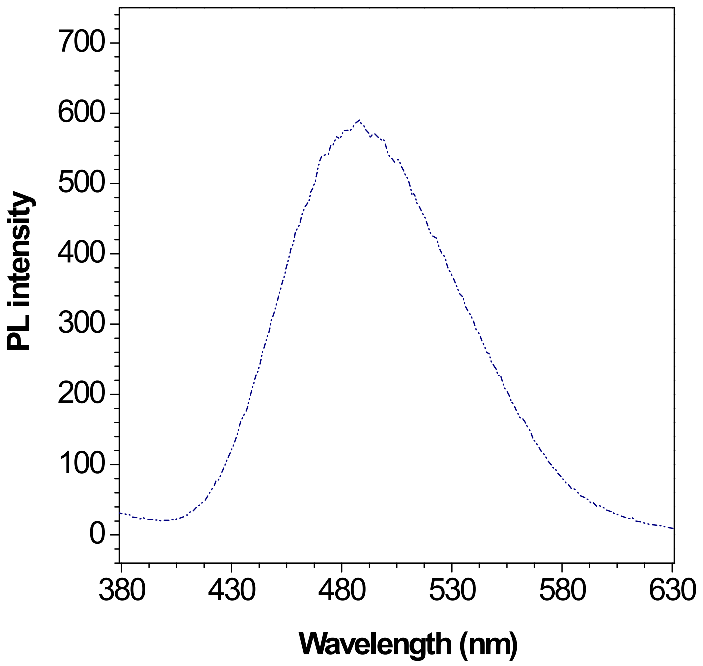

Figure S15. Photoluminescence (PL) spectrum of TPE-COOH (10 µM) in PBS buffer solution containing 1% DMSO.

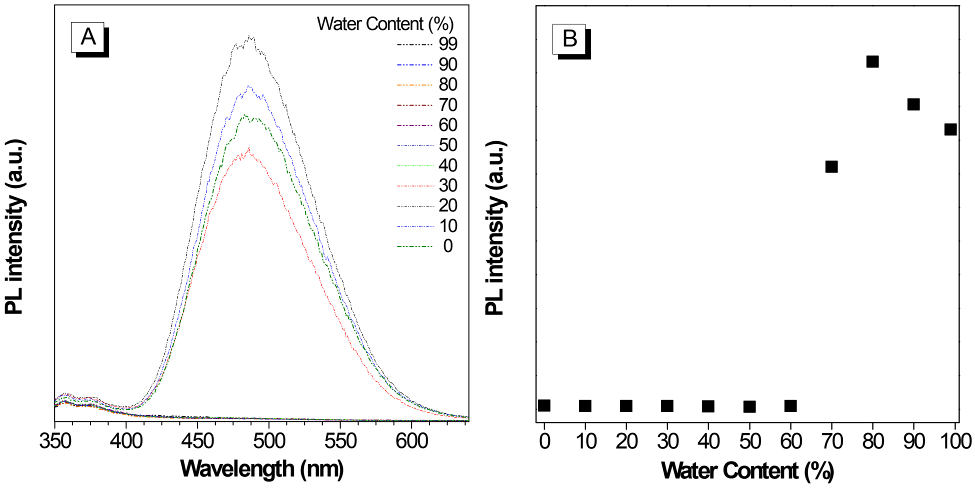

Figure S16. (A) Photoluminescence (PL) spectra of TPE-COOH in DMSO/water mixtures with different water fractions (fw). (B) Plot of peak intensity vs fw. Concentration: 10 μM; excitation wavelength: 390 nm.

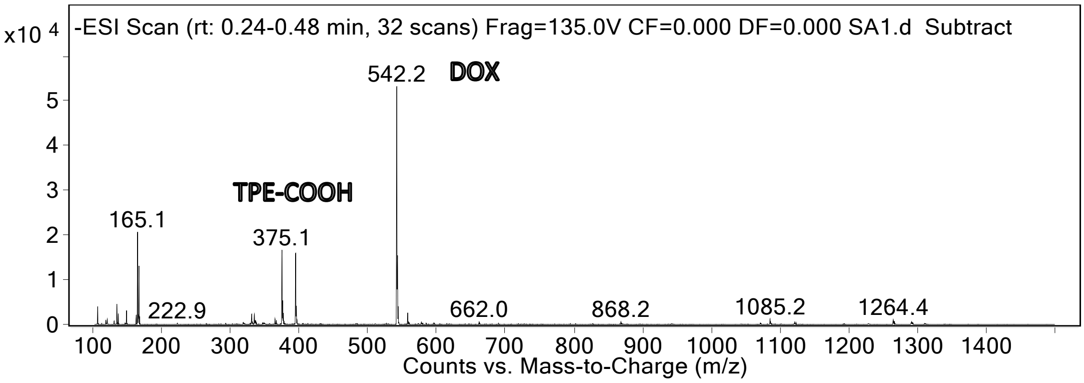

Figure S17. Mass spectrum analysis of ABD system (10 μM) incubated with H_2O_2 (100 μM) for 90 minutes.
